# Tetanus immunity in nursing home residents of Bolu, Turkey

**DOI:** 10.1186/1471-2458-5-5

**Published:** 2005-01-12

**Authors:** Oguz Karabay, Fatma Ozkardes, Ali Tamer, Kazım Karaarslan

**Affiliations:** 1 Department of Infectious Diseases and Clinical Microbiology, Izzet Baysal University, Faculty of Medicine, Golkoy, Bolu, Turkey; 2 Department of Microbiology, Izzet Baysal University Faculty of Medicine, Golkoy, Bolu, Turkey; 3 Department of Internal Medicine, Izzet Baysal University Faculty of Medicine, Golkoy, Bolu, Turkey; 4 Department of Anaesthesiology and Reanimation Izzet Baysal University Faculty of Medicine, Golkoy, Bolu, Turkey

## Abstract

**Background:**

Tetanus is a serious but vaccine-preventable disease and fatality rate of the disease is high in the neonates and the elderly. The aim of this study was to detect the tetanus antibody prevalence in the over sixty-year age residents of the nursing homes in Bolu.

**Methods:**

A voluntary-based study was done in the residents of two nursing homes in Bolu, Turkey. Blood samples were taken from 71 volunteers residing in there nursing homes. Tetanus IgG antibodies were measured by a commercial ELISA kit.

**Results:**

Among overall subjects, only 11 (15.7 %) had the protective tetanus antibody titers at the time of the study. Totally, 10 subjects were examined in emergency rooms due to trauma or accidents within the last ten years and, four (40%) of them had protective antibody levels. Of the remaining 61 subjects only 7 (11%) had protective antibody levels (p < 0.05) [Relative Risk = 3.49, 95% Confidence Interval 1.24–9.77].

**Conclusions:**

Tetanus antibody level is below the protective level in the majority of the over-sixty-year-age subjects residing in the nursing homes. Each over sixty-year age person in our country should be vaccinated. Until this is accomplished, at least, nursing home residents should be vaccinated during registration.

## Background

Tetanus is an acute disease caused by the tetanus toxin, released by the bacterium *Clostridium tetani*. Tetanus spores are present in soil and manure and may be introduced into the body through a puncture wound, burn or scratch. Tetanus can never be eradicated because the spores are widely spread in the environment, and found in the intestinal flora of domestic animals, horses, chickens, and humans. Tetanus is not spread from person to person [1].

Tetanus is preventable by proper immunization. However, the disease is still prevalent even in the most developed countries and it often emerges in the elderly [2]. The prevalence of neonatal tetanus is considered a criterion for developmental level of the country. Every year, the cases of neonatal tetanus are reported in four to six European countries; Turkey and Albania have the highest occurrence rates [3].

The mortality rate of tetanus is high in the neonates and the elderly patients [4]. Age-related mortality rates were found to be 79.4 % in the neonatal period [5], 11 % in adults younger than 50 years old and 54 % in adults older than 50 years old [4,6]. Totally, 280 cases were observed in Turkey between 1994 and 1995. The mortality rate was 29.8% for these cases [7]. Risk is greater in people over 60, particularly in the developed countries [8].

In the USA, the disease occurs at a six-times higher rate in this age group [8,9]. Of 99 tetanus patients whose complete information was reported to the Centers for Disease Control and Prevention (CDC) during 1987 and 1988, 68% were over 50, while only six were younger than 20. No cases of neonatal tetanus were reported. The disease continues to occur almost exclusively among people who are unvaccinated or inadequately vaccinated or whose vaccination histories are unknown or uncertain [10].

Bolu is a city in the Western Black Sea Region of Turkey and has a population of approximately 283.000. There are two nursing homes (Izzet Baysal and Neziha Baysal Nursing Homes) in the each with a boarding capacity of 60 people. Tetanus antibody level in such a specific group has not been researched in the country yet. The purpose of this study was to detect tetanus antibody prevalence in the voluntary residents of the nursing homes over 60 years old.

## Methods

This study was performed May 1–30, 2004. Subjects were selected from Izzet Baysal and Neziha Baysal Nursing home residents who volunteered for antibody measurement.

A standard form was filled including subjects' clinical information, demographic traits along with vaccination and injury stories. 10 cc venous blood was taken from each subject and kept at -20°C until the day of study.

Tetanus Ig G antibodies were measured by a commercial ELISA kit [Novatec Dietzenbach, Germany] at a 450/620 nm wavelength. The results were evaluated in the way previously defined by Schoroder et al. [11]. Briefly, antitoxin levels below 0.1 IU/L were defined as "below protective level" and antitoxin levels above 0.1 IU/L were defined as "at protective level".

Students't-test was used to compare quantitative variables while chi-square and Fischer's exact tests (two-tailed) were used for qualitative data, p < 0.05 was considered to be significant. Statistical analyses were performed with Epi-info 6.0 (Center for Disease Control, Atlanta, USA).

## Results

### Subjects

We aimed to include all residents of the nursing homes. In the study period, 78 subjects were staying in the two nursing homes (40 residents in Izzet Baysal Nursing Home and 38 residents in Neziha Baysal Nursing Home). However, 7 subjects did not want to participate in the study. Therefore, these seven subjects could not be included.

### Antibody results and properties of subjects

**Figure 1:**
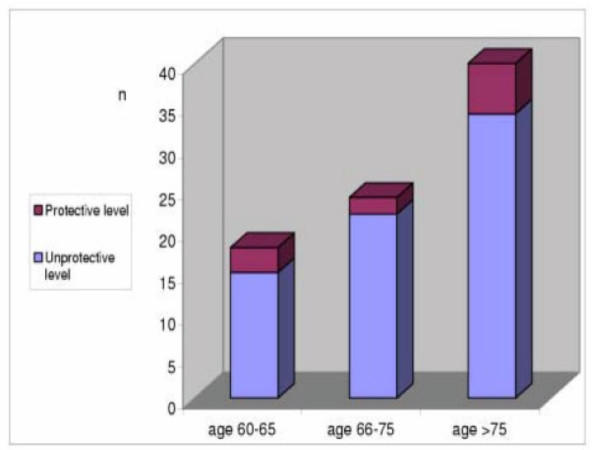
Antibody levels of subjects

A total of 71 voluntary subjects [54 (76%) male and 17 (24%) female] were included in the study. The mean age was 71. Only 11 (15.4%) of the 71 subjects had a protective antibody level against tetanus. 15 subjects were aged between 60–65 (3 of them had protective antibodies), 22 subjects were aged between 66–75 (2 of them had protective antibodies) and 34 of 71 subjects were aged ≥ 76 years (6 of them had protective antibodies). Protective antibody level decreased with every age group (60–65, 66–75, and ≥ 76) above 60 years old, but there were no statistically significant difference between the five-year age periods above 60 (p > 0.05) (Fig. 1).

Totally, 10 subjects were examined in emergency rooms due to trauma or accidents within the last ten years. While four (40%) of them had protective antibody levels, only 7 (11%) of 61 subjects who didn't get examined in the emergency room had protective antibody levels (p < 0.05) [Relative Risk = 3.49, 95% Confidence Interval 1.24–9.77].

Only 10 (14%) of the 71 subjects had been vaccinated in the last ten years. Five of them had been vaccinated as a result of emergency room visits. Antibody level and demographic properties of subjects were presented in the Table 1.

**Table 1:** Demographic characteristics and antibody levels of subjects according to age groups

Characteristics	TOTAL n = 71 (%)
Age (mean ± SD)	71.1 ± 8.6
Female/Male	17/54
Diabetes mellitus	7 (9.8)
Emergency room visits due to injury within the last 10 years	10 (14)
Tetanus vaccine within last 10 years Emergency room visit/No emergency room visit	5 (7)/5 (7)
Occupation with soil / garden-work	20 (28.1)
Average tetanus Ig G level	0.080
Tetanus Ig G >0.1 IU/ml	11 (15.4)

## Discussion

According to the records of the Ministry of Health, 2039 tetanus cases were detected in Turkey between 1980–2002, 462 (23%) of which died [12]. In our country, a vaccination campaign against tetanus was initiated in the mid 1960s. However, it was carried out irregularly until 1985. After 1985, 3 doses of tetanus toxoid were given to all neonates after birth and a booster dose was applied in the 16th month followed by vaccination of primary school children at aged 7 and 12. While 67 neonatal tetanus cases were detected in 1990 in a population of 57.582.244, 32 neonatal tetanus cases were detected in 2002 in a population of 70.415.244 [12]. The neonatal tetanus rate has decreased with an increased rate vaccination in the country. In Turkey, females are vaccinated during pregnancy and males are vaccinated during military service but there is no vaccination program for the elderly. Unfortunately, a falling could not be achieved in the prevalence of tetanus in the elderly. Similar results were obtained in the developed countries, too. For instance, it was established that, rate of occurrence of tetanus in the people older than 60 was ten times higher than the young in Italy [9]. Routine vaccination programs focus on the childhood period in the developing countries. However, according to researches, the majority of adult population is sensitive to tetanus [13].

In our study group, only 11 (15.4%) of 71 subjects had protective antibody levels while the rest were under the risk of tetanus. This resultsuggested that lack of protective immunity among nursing home residents was a significant public health concern. In industrialized countries tetanus has become a rare disease and an infrequent cause of death, mainly due to the implementation of comprehensive immunization programs. But, tetanus is still an important problem for the developing countries, due to poor immunization standards and inadequate hygiene [14]. Deaths due to tetanus in Turkey mostly occur in the elderly. The 27.4 % of the total deaths caused by tetanus in 1989 occurred in the patients over 40. But today mortality rate in this age group ranges between 48.8–60.6% [15]. Those who are not vaccinated and the elderly are at risk [16].

Individuals over 60 are usually retired people and spending most of their time in garden or land-work. Tetanus spores may be introduced into the body through a puncture wound, scratch during these works. So they face risk of getting tetanus. Also, 28 % of our subjects still were busy with soil and garden work.

According to our results, antibody levels in individuals who had been examined in an emergency room within the last ten years due to injury are significantly higher than who didn't have such a history (p < 0.05). This situation suggests that vaccination programs are not well established in countries like Turkey, thus injuries and accidents expose people over 60 to a great risk of getting the tetanus prophylaxis [17].

Once the existence of tetanus is suspected, intensive, and effective management is essential. The patient should receive intensive care aimed at prevention of muscle spasms, prevention of respiratory tract and metabolic complications, and neutralization of circulating toxins [18]. Assuming that one tetanus case stays in the intensive care unit for at least fifteen days, it costs $9.000 per case approximately. In Turkey, 30.000 people can be vaccinated with a booster dose of tetanus vaccine for this amount of money [19,20]. Moreover, vaccination will not only ensure economic benefits but also protect thousands of people against tetanus. Apart from being more profitable, such policy will improve the health statistics of the country [20]. It is imperative that every person over 60 should be vaccinated against tetanus.

## Conclusions

The lack of protective immunity against tetanus among the nursing home residents is a significant public health concern. A vaccination program including every individual over 60 should be charted immediately. After the primary series of 3 doses, protection against tetanus should be sustained by scheduling booster doses routinely in every 10 years [21]. Until this campaign is accomplished, at least, nursing home residents should be vaccinated during registration.

## Competing interests

The author(s) declare that they have no competing interests.

## Authors' contributions

All authors read and approved the final manuscript. OK designed the study and drafted the manuscript. AT, KA, analysed the data. OK oversaw the microbiological research. OK and FO interpreted the results of the analysis and critically reviewed the final manuscript.

## Pre-publication history

The pre-publication history for this paper can be accessed here:


